# T cell co-stimulatory and co-inhibitory pathways in atopic dermatitis

**DOI:** 10.3389/fimmu.2023.1081999

**Published:** 2023-03-13

**Authors:** Chunjiao Zheng, Yuling Shi, Ying Zou

**Affiliations:** ^1^ Skin and Cosmetic Research Department, Shanghai Skin Disease Hospital, Tongji University School of Medicine, Shanghai, China; ^2^ Institute of Psoriasis, Shanghai Skin Disease Hospital, Tongji University School of Medicine, Shanghai, China

**Keywords:** atopic dermatitis, T cells, co-signaling molecules, co-stimulatory molecules, co-inhibitory molecules

## Abstract

The use of immune checkpoint inhibitors (ICIs) targeting the T cell inhibitory pathways has revolutionized cancer treatment. However, ICIs might induce progressive atopic dermatitis (AD) by affecting T cell reactivation. The critical role of T cells in AD pathogenesis is widely known. T cell co-signaling pathways regulate T cell activation, where co-signaling molecules are essential for determining the magnitude of the T cell response to antigens. Given the increasing use of ICIs in cancer treatment, a timely overview of the role of T cell co-signaling molecules in AD is required. In this review, we emphasize the importance of these molecules involved in AD pathogenesis. We also discuss the potential of targeting T cell co-signaling pathways to treat AD and present the unresolved issues and existing limitations. A better understanding of the T cell co-signaling pathways would aid investigation of the mechanism, prognosis evaluation, and treatment of AD.

## 1 Introduction

Atopic dermatitis (AD or atopic eczema) is an immune-mediated chronic inflammatory skin disease with recurrent eczema lesions and intense itching (1). AD is one of the most common inflammatory diseases, where it affects approximately 20% of children and 10% of adults in high-income countries (2, 3). AD prevalence is increasing by the year and can occur at all ages and ethnicities; it is typically common in early childhood and in adulthood (4, 5). The disease exerts a serious social and psychological effect on patients and relatives and is the key reason for the global burden of skin diseases. The etiology of AD is complex and contains strong genetic components, and environmental factors (6). AD patients are prone to asthma, allergic rhinitis, or food allergy, and face an increased risk of adverse psychological diseases (7). In most patients, AD is a lifelong disease with clinical heterogeneity and multifactorial pathogenesis. Innovative biological and small molecular therapies can be targeted to treat the pathogenesis mediated by epidermal barrier dysfunction and type 2 skin inflammation (6). Cutaneous inflammation is central to AD pathogenesis. A better understanding of the key drivers of AD is important to develop targeted therapeutic approaches.

The lesional skin of patients with AD exhibits a predominantly helper type 2 (Th2) cell infiltrate (6). The inflammatory profile is complex and diverse, with activation of skin-resident inflammatory dendritic cells (DCs), innate lymphoid cells, and Langerhans cells (LCs) (8). The release of alarmins triggered by epidermal barrier disruption activates inflammatory DCs and type 2 immunity responses (**Box 1**) (6, 9). In AD, Th2 dominant inflammation is characterized by CD4^+^ T cells and eosinophil (Eos) infiltration into the dermis, with Eos deposition and increased Th2 cytokine expression in the skin (6). Activated Th2 cells release interleukin (IL)-4 and IL-13, promoting B cell IgE class switching and producing antigen-specific IgE *via* the signal transducer and activator of transcription (STAT) pathway (9). The high- affinity IgE receptor (FcϵRI) in mast cells (MCs) combines with IgE, resulting in FcϵRI starting the signal cascade reaction, which induces calcium mobilization, leads to MCs degranulation, and promotes immediate hypersensitivity (12). Given the important role of Th2 cells in AD inflammatory mechanisms, the adaptive immune system and activation pathways involved in Th2 cells are of particular interest. T cell activation is a complicated and carefully regulated process. Following the stimulation of naive T cells, non-effector T cells differentiate and migrate to the affected area. If a second infection occurs, the subsequently formed memory T cells would have acquired the ability to respond more quickly (13, 14). Activated T cells can pass through the JAK–STAT (Janus kinase–signal transducers and activators of transcription) (**Box 2**), PI3K–AKT–mTOR (phosphoinositide 3-kinase–AKT–mammalian target of rapamycin), TGF (transforming growth factor), NF-κB (nuclear factor κB), PPAR (peroxisome proliferator-activated receptor), and other intracellular signal pathways that affect their proliferation, differentiation, effects, and memory functions (31–33). It is well known that co-inhibitory and co-stimulatory signals regulate T cell activation, but understanding of the role of signaling pathways in AD remains incomplete. With the rising use of immune checkpoint inhibitors (ICIs) targeting these pathways in cancer therapy, a timely overview of the role of the co-signaling pathways in AD is necessary.

**Box 1 Type 2 immunity**

Type 2 immunity is one of three types of innate and adaptive immune systems that primarily target large extracellular parasites. Alterations in type 2 immune responses are closely related to allergic diseases (9). Type 2 immune responses begin at the epithelial interface, where triggers such as thymic stromal lymphopoietin, IL-25, and IL-33 activate Th2 cells, group 2 innate lymphoid cells, and B cells (9, 10). The type 2 immune response effectors include IgE and effector cells such as Eos, basophils, and MCs (9). Activated Th2 cells and Th22 cells secrete IL-4, IL-5, IL-9, IL-13, and IL-31 (10, 11). Excessive and chronic activation of the above pathways leads to atopic diseases.

 

**Box 2 The JAK–STAT pathway in AD**

The JAK family includes JAK1–3 and tyrosine kinase 2 (TYK2), which are linked to the intracellular domains of multiple transmembrane cytokines (15). The JAKs are activated and phosphorylated after selectively binding to the receptor. JAK function is coupled to the STAT family (16), which includes seven intracellular transcription factors (STAT1–4, 5A, 5B, 6) (17). STATs translocate to the nucleus after phosphorylation, regulating gene transcription (16). The JAK–STAT pathway is crucial for the downstream signal transduction of inflammatory cytokines, including interleukins, interferons, and various growth factors (18). All JAKs and STATs are involved in the AD inflammatory process (18, 19).

Upon binding to its corresponding receptor, the inflammatory factor IL-4/IL-13 activates STAT3 or STAT6 through JAKs and regulates T cell proliferation and Th2 differentiation by upregulating GATA3 expression (20). IL-5 transduces signals through JAK1/2 and STATA1/2/5 (21). IL-31 and TSLP promote Th2 differentiation by activating STAT1/3/5 *via* JAK1/2 (22, 23). After IL-22 binds to the receptor, JAK1 and TYK2 phosphorylate and activate STAT1/3/5, which is key in skin modification and epidermal proliferation (24, 25). IFN-γ and IL-12 are important in Th1 differentiation *via* STAT1 and STAT4 transduction, respectively (26, 27). IL-17 affects Th17 differentiation by inducing retinoic acid receptor-related orphan receptor γ (RORγt) expression *via* STAT3 (26). STAT6 inhibits FOXP3 by upregulating GATA6, affecting Treg differentiation, proliferation, and maintenance (22, 28). The JAK–STAT signal pathway also affects Eos proliferation, survival, and function (21, 29), and is involved in MC homeostasis and proliferation in AD (30).

The classical 2-signal model suggests that T cells must undergo antigen and secondary stimulation to induce activation (34, 35). Major histocompatibility complex (MHC) peptide complex binding to T cell receptor (TCR) produces the first signal to activate T cells, but this activation is insufficient and T cells may only be partially activated or even unresponsive (36). Further activation requires a second signal, i.e., the co-signaling molecule on T cells, which binds to the corresponding ligand of the antigen-presenting cells (APCs) so that the T cells can be fully activated and perform their optimal immune function (37, 38).

Several other co-signaling molecules were identified following the discovery of the T cell-specific surface glycoprotein CD28 (39). Most of these molecules are members of the immunoglobulin superfamily (Ig-SF) and tumor necrosis factor receptor superfamily (TNFR-SF) (31), while some belong to the integrin superfamily (Integrin-SF) (40). Co-signaling molecules can be subdivided into specific families based on their primary amino acid sequence, protein structure, and function (31, 40). Excessive co-stimulation or inadequate co-inhibition can lead to abnormal T cell activation, which results in the breakdown of T cell tolerance (31).

Immune checkpoint regulators (ICRs) can be either stimulatory or inhibitory molecules. The introduction of ICIs revolutionized the treatment of certain cancers and greatly improved survival rates. ICIs affect the balance between T cell activation and suppression by reducing inhibitory signals or enhancing stimulatory signals, thereby increasing cancer cell clearance (41).

The unusual expression of co-stimulatory and co-inhibitory signaling pathways has been reported in various autoimmune diseases, such as systemic lupus erythematosus (42), rheumatoid arthritis (42), multiple sclerosis (42), type 1 diabetes (42), psoriasis (43), and allergic diseases, such as asthma (44), which indicated the possibility that ICR treatment might contribute to AD development, which has been excellently reviewed elsewhere (45–47). Some ICIs are used in clinical settings and many more are in clinical trials for treating various cancers with unknown effects and degrees of risk on cutaneous immune-related adverse events (irAEs) (48). It was recently reported that the co-signaling molecules in immune cells are involved in AD pathogenesis and present prospects for new therapies (44, 49).

**Figure 1:**
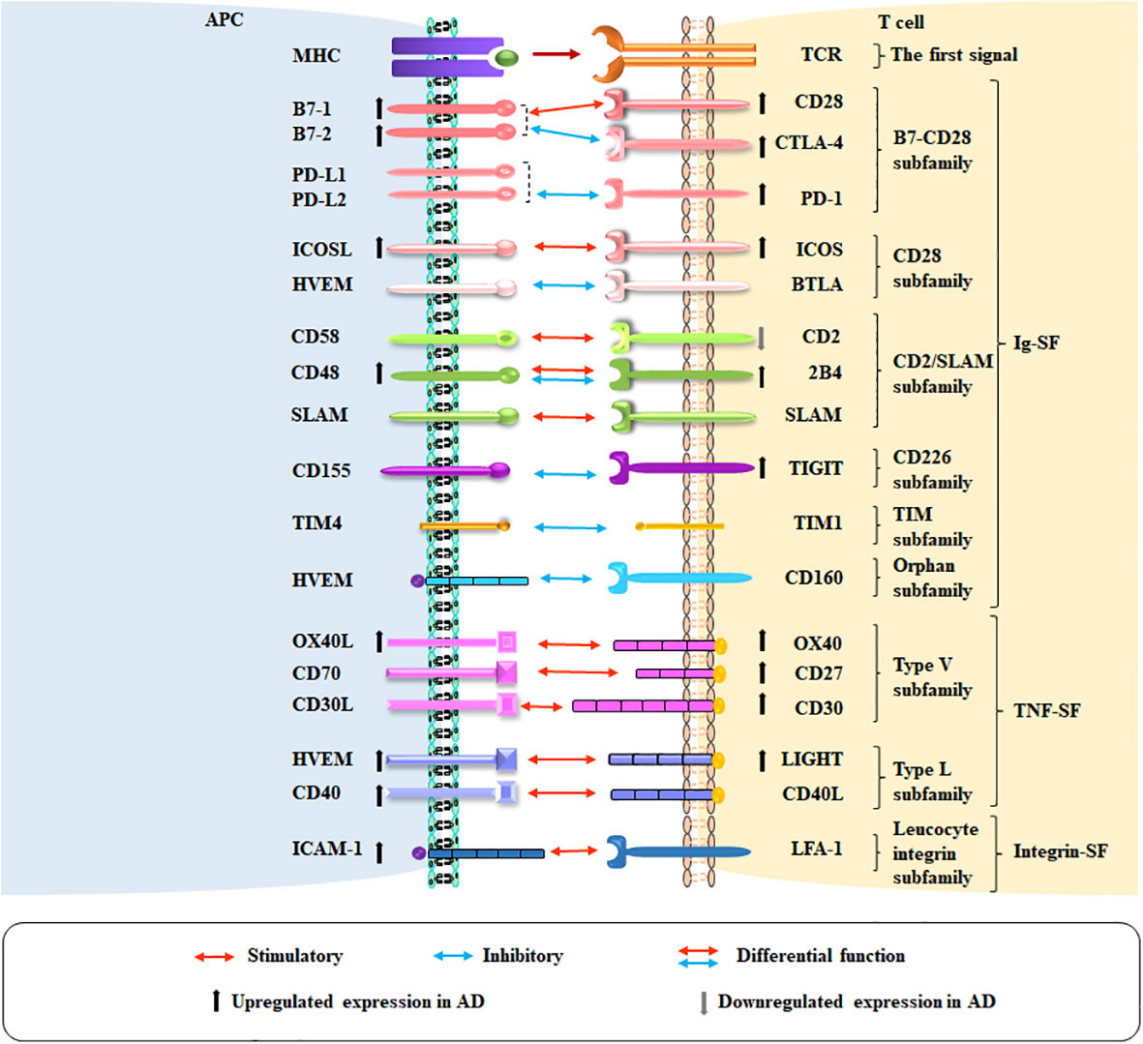
Co-stimulatory and co-inhibitory pathways in AD. The co-stimulatory and co-inhibitory molecules on APCs and T cells in AD are shown. Red arrows indicate co-stimulation, blue arrows indicate co-inhibition, and red and blue arrows together indicate molecules with co-inhibitory and co-stimulatory functions. Ascending and descending arrows indicate the increase and decrease of co-stimulatory and co-inhibitory molecules in AD, respectively.

**Figure 2:**
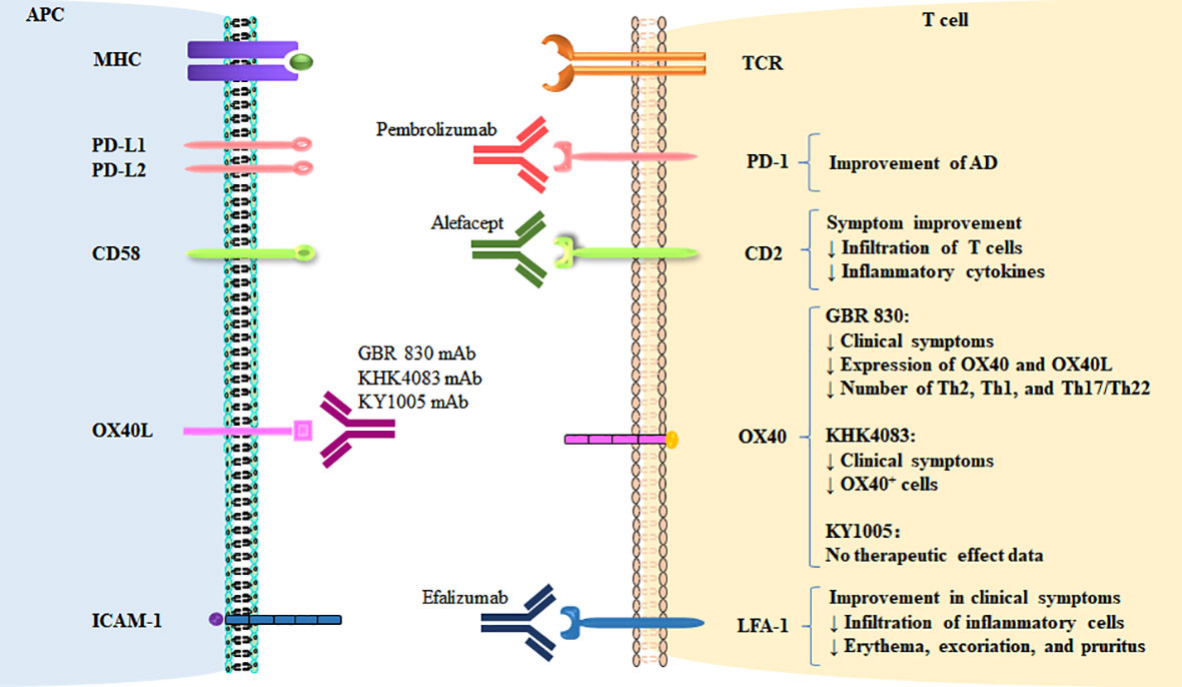
Therapeutic targeting of co-stimulatory and co-inhibitory pathways in AD. The targeting of co-signaling molecules in AD and therapeutic effects are shown.

In this review, we highlight and put into perspective the current knowledge on T cell co-signaling pathways and the role of these pathways in AD (**Figure 1**). We summarize the available preclinical and clinical data on the differential functions of the Ig-SF, TNF-SF, and Integrin-SF co-signaling molecules and their opposing roles in AD (**Figure 2**, **Tables 1**–**3**). We also discuss potential future therapeutic options aimed at affecting the co-signaling pathways to alter T cell responses and treat AD. This information might contribute to better understanding of the mechanism and guide future treatment for AD.

**Table 1:** Preclinical and clinical studies on T cell co-stimulatory and co-inhibitory molecules of the Ig-SF.

Molecule on APC	Receptor on T cell	Experimental model	Effects on AD	Related cytokines	Perspectives/limitations	Ref.
B7-CD28 subfamily						
CD80/CD86	CD28	DNFB-treated mice	↑ CD28/CD80/CD86 protein expression and positive cells	IL-8, IL-12, IL-23	▪ Few studies ▪ Anti-CTLA-4 might result in irAEs	50
		▪ DNFB-treated mice ▪ HLJDT treatment	↓ CD28/CD80/CD86 protein expression and positive cells ↓Clinical score ↓Ear swelling			50
		Skin lesions of AD patients	↑Expression of CD80/CD86 on LCs			51, 52
		Serum samples of AD patients	↑Expression of CD28 autoantibodies			53
		▪ PBMCs of AD patients ▪ Anti-CTLA-4 treatment	↑Proliferation and IgE synthesis of PBMCs			54
	CTLA-4	NC/Nga mice (application of mite antigens)	↑*CD80*/*CD86*/*CTLA4* gene expression in the skin			55
		▪ NC/Nga mice (application of mite antigens) ▪ La1 treatment	↓CD80/CD86/CTLA-4 expression ↓Development of AD-like lesions ↓Skin score			55
		AD infants	CTLA-4 overexpression			56
		Peripheral blood of infants with moderate to severe AD	↑CTLA-4 express on Tregs			57, 58
PD-L1/PD-L2	PD-1	IL-4 transgenic mice	↑PD-1 expression on CD4^+^ and CD8^+^ T cells	–	Block PD-1 or PD-L1 might result in irAEs	59
		▪ MC903-treated PD-L1^-/-^ mice ▪ MC903-treated PD-L2^-/-^ mice	↑Ear swelling in PD-L1^-/-^ mice ↑Ear swelling in PD-L2^-/-^ mice			60
		▪ NC/Nga mice ▪ PD-L2 siRNA treatment	Ineffective			61
		PBMCs of infants with moderate to severe AD	↑PD-1 expression on Tregs			62
		▪ AD patients with disseminated Kaposi sarcoma ▪ Treatment with pembrolizumab	AD improved			63
CD28 subfamily						
ICOS	ICOSL	VitD mouse model of AD	↑Percentages of ICOS^+^ Tregs in sdLNs	IL-10	Studies focus on Tregs	64
		PBMCs of AD patients	↑Percentages of ICOS^+^ Tregs ↓Production of IL‐10			65
		PBMCs and lesional skin of Han Chinese population with AD	↑ICOS^+^ Th22 ICOS^+^ Th22 and ICOSL^+^ B cells were related to disease activity and total serum IgE levels ↑Expression of ICOS and ICOSL expression in lesional skin			66
		DNA and blood samples of European children	None of the six evaluated *ICOS* gene variants was related to the development of allergic phenotypes			67
HVEM	BTLA	▪ CXCR5^hi^PD1^hi^ CD4 naive T (Tfh characteristics) co-culture with TSLP-activated DCs ▪ AD donors	↑BTLA protein and mRNA ↑Tfh in AD	–	Need more studies	68
CD2/SLAM subfamily						
CD58	CD2	PBMCs of AD patients in children	↓CD2^+^ lymphocytes	IL-5, IL-10, IL-13, IFN-γ, GM-CSF	No biological targets currently	69
		▪ PBMCs of AD patients ▪ Anti-CD2-blocking mAb treatment	↓INF-γ, GM-CSF, and IL-5 levels			70
		▪ AD patients ▪ Treatment with alefacept	Symptom improvement ↓Skin T cells ↓IL-5, IL-10, IL-13, IFN-γ			71
CD48	2B4	▪ Eos of AD patients ▪ SA exotoxin treatment	↑CD48^+^ Eos	–	Need more studies	72
		2B4^-/-^ mice induced with 2-week OVA/SEB	↑Eos trafficking			73
		2B4^-/-^ mice induced with 3-week OVA/SEB	↑MC activation			74
		Peripheral T cells of AD patients	↑*2B4* gene expression			75
SLAM	SLAM	▪ Th2 cell populations derived from skin biopsies of AD patients ▪ Treatment with SLAM by an agonist mAb	↑INF-γ-producing cells	IFN-γ	Reverse the Th cell phenotype of AD	76
CD226 subfamily						
CD155	TIGIT	PBMCs of AD patients	↑TIGIT expression The proportion of TIGIT^+^ cells was correlated with AD severity The frequency of TIGIT^+^ cells in CD4^+^ T cells was negatively correlated with serum thymus and activation-regulated chemokine levels and lgE levels in AD patients	–	Need more evidence on TIGIT functions	77
TIM subfamily						
TIM4	TIM1	▪ OVA-sensitized AD-like mouse model ▪ AD-like skin lesions of NC/Nga mice	TIM4 expression on Langerhans-like DCs inhibited Th2 cell development and was beneficial for controlling AD	–	Need further research	78–80
		Blood samples of AD patients	TIM1 exon 4 variations were associated with AD			81
		Australian Caucasian families and Asian families	A novel association between AD and the major haplotype of TIM4 Genetic variants in the ligand for TIM1 and TIM4 contributed to AD presentation			82
Orphan subfamily						
HVEM	CD160	Skin lesions of AD patients	↑CD160^+^ T cell infiltration The engagement of CD160 enhanced the CD4^+^CD160^+^ cell proliferation induced by CD3 stimulation	–	Need experiments to elucidate the exact functions	83

APC, antigen-presenting cell; CTLA4, cytotoxic T lymphocyte antigen 4; PD-1, programmed cell death 1; PD-L1/PD-L2, programmed cell death 1 ligand 1/2; ICOS, inducible T cell co-stimulator; ICOSL, ICOS ligand; BTLA, B and T lymphocyte attenuator; HVEM, herpesvirus entry mediator; SLAM, signaling lymphocyte activation molecule; TIM, T cell immunoglobulin and mucin domain.

**Table 2:** Preclinical and clinical studies on T cell co-stimulatory and co-inhibitory molecules of the TNF and TNFR superfamilies.

Molecule on APC	Receptor on T cell	Experimental model	Effects on AD	Related cytokines	Perspectives/limitations	Ref.
Type V subfamily						
OX40L	OX40	*In vitro* cell study	OX40L DCs trigger Th2 differentiation	TSLP, IFN-γ, CXCL10, IL-31, CCL11, CCL17, TSLPR, IL-23p19, IL-8	Phase 2 clinical trials achieve promising results	84
		Lesional skin of AD patients	↑OX40L^+^ DCs			85, 86
		Skin of AD patients	↑ OX40 expression on skin-homing T cells Co-localization of OX40 and OX40L on skin MCs			87
		▪ Skin lesions of AD patients ▪ Treatment with GBR 830	↓OX40 T-cell ↓OX40L DCs ↓Th2, Th1, Th17, Th22 cell numbers ↓Expression of IFN-γ, CXCL10, IL-31, CCL11, CCL17, TSLPR, IL-23p19, IL-8, S100A			88
		▪ AD patients ▪ Treatment with KHK4083	↓OX40^+^ cells Continued improvements in eczema area, severity index, and global assessment scores			89
		▪ AD patients ▪ Treatment with KY1005	No therapeutic effect data			90
CD70	CD27	MC903-treated C57BL/6 mice	↑CD27 expression on LCs	IL-12	Require more studies	91
		AD patients	↑CD27 expression on CD4^+^ T cells			92
CD30L	CD30	Serum samples of AD patients	↑sCD30 sCD30 levels were positively correlated with AD disease severity	–	Lack animal model validation and relevant research on blocking antibodies	93–107
		Serum samples of AD patients	↓sCD30 levels after treatment			93, 95, 98, 103, 107
		Serum samples of AD patients	The sCD30 concentration was correlated with the disease activity and total serum IgE			101–103, 108
Type L subfamily						
HVEM	LIGHT	Human KCs	LIGHT directly promoted TSLP expression in KCs	TSLP	A promising biomarker for AD treatment	109
		LIGHT-deficient mice and K14-cre HVEM mice	↓Clinical symptoms			110
		Human KCs	LIGHT promoted KC proliferation and was prevented by siRNA knockdown of HVEM			110
		▪ HDM-sensitized C57BL/6 mice ▪ Anti-HVEM antibody treatment	↓SCORAD ↓Epidermal and dermal thickness ↓Periostin and TSLP expression			110
		Serum samples of AD patients	↑sHVEM and LIGHT levels			111
		Plasma samples of AD patients	Plasma LIGHT concentrations correlated with IgE levels and SCORAD, decreased as the symptoms were improved by treatment			112
CD40	CD40L	Peripheral blood of AD patients	↑CD40L on T cells	–	Target the pathway may benefit AD patients	113
		Skin lesions and peripheral blood of AD patients	↑CD40^+^ cells CD40^+^ cells as a positive correlation with disease severity and lgE levels			114
		PBMCs of AD patients	↑CD40 expression on B cells			114

APC, antigen-presenting cell; OX40L, OX40 ligand; CD30L, CD30 ligand; LIGHT, homologous to lymphotoxin, a receptor expressed on T lymphocytes, also known as HVEM-L; CD40L, CD40 ligand.

**Table 3:** Preclinical and clinical studies on T cell co-stimulatory and co-inhibitory molecules of the integrin superfamilies.

Molecule on APC	Receptor on T cell	Experimental model	Effects on AD	Related cytokines	Perspectives/limitations	Ref.
Leucocyte integrin subfamily						
ICAM-1	LFA-1	▪ KCs stimulated with TNF-α and IFN-γ *in vitro* ▪ UV-LED treatment	↓ICAM-1 expression	IL-4, IFN-γ	▪ Show promise for treating AD ▪ Need more clinical studies for further testing	115
		▪ NC/Nga mice ▪ Anti-LFA-1 mAb treatment	↓Skin lesion development ↓IgE and lymphocyte cytokine production			116
		Lesional skin of chronic and acute AD patients	↑ICAM-1 expression			117
		Lesional skin of AD patients	↑ICAM-1 expression on KCs ICAM-1 was not a suitable marker of actual disease activity			118
		Serum samples of AD patients	↑sICAM-1 level			119–121
		▪ Severe intractable AD in children ▪ IVIG treatment	↓sICAM-1 level ICAM-1 level may be useful for monitoring disease activity of AD in childhood			122
		▪ AD patients ▪ Tacrolimus treatment	↓ICAM-1 expression			123
		▪ AD patients ▪ Treatment with efalizumab	Symptom improvement ↓Skin inflammatory cell infiltration ↓Erythema, excoriation, and pruritus			124–127

APC, antigen-presenting cell; LFA-1, lymphocyte function-associated antigen-1; ICAM-1, intercellular cell adhesion molecule-1.

## 2 The Ig-SF

The Ig-SF is represented by the presence of Ig homology domains, which are the largest and most diverse superfamily proteins found in humans (128). The Ig-SF contains many subgroup families that are expressed on the cell surface, bind diverse ligands, and contribute to various cellular activities, including adhesion and immune responses (129).

### 2.1 B7–CD28 subfamily: CD28, CTLA-4, and CD80/CD86

CD28 was the first co-stimulatory molecule discovered (39). CD28 interactions with its ligands CD80 (B7-1) and CD86 (B7-2) remain the best-characterized pathway (130). Without CD28, co-stimulatory TCR signaling often induces an anergic state or cell death (131). CD80 and CD86 are transiently expressed on APCs upon activation (132–134). CTLA-4 (CD152) is homologous to CD28 but has opposite functions as a co-inhibitory receptor to suppress the T cell response (135). CD80/CD86 also binds to the CTLA-4 expressed by activated T cells. CTLA-4 and CD80/CD86 interact with much higher affinity (10–20 times) than CD28, which contributes to T cell response regulation (136, 137). CD28 is constitutively expressed on all murine T cells but appears on only 80–95% of CD4^+^ T cells and 50% of CD8^+^ T cells in humans (138, 139). CTLA-4 is expressed largely on activated and regulatory T cells (Tregs) (140). CD28–B7 signals are critical for T cell activation, proliferation, and survival following T cell interaction with APCs presenting their cognate antigens (39, 141).

#### 2.1.1 Cell culture/animal model studies

An AD-like animal model of mice treated with 2,4-dinitrofluorobenzene (DNFB) (All AD animal models mentioned in this article are briefly explained in **Box 3**) exhibited significantly increased CD28 and CD80/CD86 protein expression and CD28^+^ and CD80/CD86^+^ cells (50). The protein expression and positive cells, clinical score, and ear swelling were significantly reduced after the application of 12.8 g/kg Huanglian Jiedu decoction (HLJDT) (50). Furthermore, Inoue et al. reported that repeated application of mite antigens in NC/Nga mice enhanced *CD80*, *CD86*, and *CTLA4* gene expression in the skin, while *CD28* gene expression was not statistically different from that of the controls (55). In young mice, Lactobacillus johnsonii NCC 533 (La1) treatment, was followed by significantly reduced *CD80*, *CD86*, and *CTLA4* gene expression, which markedly inhibited the development of AD-like lesions, reduced the skin score, and impeded the overexpression of proinflammatory factors (including IL-8, IL-12, and IL-23). Nevertheless, there was no group difference in *CD28* gene expression (55). Theoretically, the immune response in the AD-like inflammation NC/Nga mouse group should have been suppressed. On the contrary, this phenomenon might be explained by another role of CTLA-4 in AD or drastically increased T cell numbers (56, 150, 151). As described above, Inoue et al. suggested that the CTLA-4 overexpression in the AD-like NC/Nga mouse group may have been associated with skin lesion development. After treatment, gene expression might have been attenuated by the reduced CD80/CD86 co-signaling.

**Box 3 Animal model in AD.**

DNFB mice (142, 143): Repeated use of DNFB stimulation induces skin barrier alterations and Th2-biased immune responses. Long-term hapten application induces human AD-like skin lesions.

NC/Nga mice (144, 145): A spontaneous mouse model of AD. Chromosome 9 mutations are associated with IgE production and increased Th2 response. Skin changes are secondary to exposure to various environmental allergens. Erythema and erosions develop rapidly after the onset of scratching behavior at 6–8 weeks, with edema and bleeding on the ears, face, neck, and back. In the chronic phase, it manifests as a Th1 response, tissue remodeling, increased collagen deposition, dermal thickening, and increased MC numbers. These features are highly similar to that of human AD.

IL-4 transgenic mice (144, 146): Transgenic mice overexpressing IL-4 exhibit spontaneous pruritus and chronic dermatitis at 4 months of age. Such mice have elevated IgE and IgG1 levels and increased T-cell infiltration in the dermis and epidermis. The chronic lesions changes are similar to that of human AD.

MC903 mice (142, 147): The 1α,25-(OH)_2_D_3_ analogue MC903 (calcipotriol) is applied to the ear or back of the mice, which causes an AD-like syndrome characterized by redness, scaling, swelling, scabbing, and frequent scratching. Histologically, epidermal hyperplasia and infiltration of numerous inflammatory cells are observed. This model relies on epidermal TSLP expression. The TSLP–TSLPR pathway produces Th2 cytokine-associated skin inflammation. MC903 induces CD4 lymphocyte activation, increases Eos, basophil, MC, and DC infiltration in the skin, and increases Th2 cytokine secretion.

VitD mice (147): VitD causes AD-like skin inflammation similar to that of the MC903 model. TSLP significantly increases in skin lesions and induces significant CD4^+^ T cell, DC, MC, and Eos infiltration.

OVA/SEB mice (148): This model features Th2-type skin inflammation, increased CD4^+^ T and CD8^+^ T cell infiltration, and markedly increased Th-related chemokines in exposed skin.

OVA mice (142, 144): This model is induced by OVA to sensitize the skin with repeated adhesive tape peeling of the epidermis. OVA sensitizes the epidermis as an allergen, triggering a Th2-type immune response and inducing a human AD-like animal model. The mice demonstrate increased scratching behavior, epidermal and dermal thickening, increased CD4^+^ T cell and Eos infiltration, and increased expression of the Th2 cytokines IL-4, IL-5, and IL-13. Serum OVA-specific IgG1, IgE, and IgG2a are systemically elevated.

HDM mice (144, 149): Similar to the OVA model, BALB/c mice are sensitized by repeated application of recombinant mite allergen. The model demonstrates dermatitis, epidermal hyperplasia, and spongiform disease with a predominant Th2 response and increased CD4 and CD8 cell infiltration.

#### 2.1.2 Human studies

CD80/CD86 is highly expressed on LCs in the skin lesions of patients with AD (51, 52). The presence of CD28 autoantibodies in the serum samples of patients with AD was highly significantly associated with AD (53). High B cell expression of CD80/CD86 with anti-CD40 and IL-4 was reported in the peripheral blood mononuclear cells (PBMCs) of patients with AD (54). Based on this discovery, Oberwalleney et al. observed markedly enhanced PBMC proliferation and IgE synthesis in the AD group in the presence of anti-CTLA-4 (54). Furthermore, Choi et al. demonstrated CTLA-4 overexpression in AD infants as compared to healthy controls (56). Jones et al. reported that the *CTLA4* gene contained several polymorphisms, some of which contributed to AD development in infants (151). CTLA-4 was also highly expressed on the Tregs in the peripheral blood of infants with moderate to severe AD, where CTLA-4 expression levels indicated the Treg suppressive efficacy (57, 58). Therefore, more Tregs were activated in moderate to severe AD and had higher suppressive potency.

The above preclinical findings revealed that reducing either the B7–CD28 co-stimulatory pathway or the B7–CTLA-4 co-inhibitory pathway had beneficial effects on AD. Accordingly, B7–CD28/CTLA-4 blockade may be a possible AD treatment. Based on the higher affinity of CTLA-4 binding to CD80/CD86 than CD28, the development of CTLA-4–CD80/CD86 blockade appears to be a more definitive approach. As expected, the numerous studies were followed by the development of various molecules to target the CTLA-4–CD80/CD86 signaling pathway, and certain clinical therapeutic effects were achieved (152–154). The ICI ipilimumab, which blocks CTLA-4, has been used clinically in anti-cancer therapy and demonstrated good therapeutic effects. However, the co-inhibitory pathway blockade enhanced T cell activation and immunosuppressed the Treg-dependent pathways, which resulted in irAEs (155, 156), among which eczema is common. CTLA-4–CD80/CD86 signaling pathway targeting has been studied in various skin diseases (157–159).

Currently, there are few studies on the CTLA-4–CD80/CD86 signaling pathway in AD, and the specific mechanism of many pathways remains unclear. More preclinical evidence on B7–CD28/CTLA-4 function should be obtained before concluding that these molecules are a potential clinical target for treating AD.

### 2.2 B7–CD28 subfamily: PD-1, PD-L1, and PD-L2

The B7–CD28 family also includes the co-inhibitory receptor PD-1, which is expressed by T cells and was discovered in 1992 as an upregulated gene in T cell hybridomas that undergo cell death (160, 161). The PD-1 ligands are PD-L1 (B7-H1 or CD274) and PD-L2 (B7-DC or CD273). PD-1 binding to the ligands is critical in T cell activation, tolerance, and immune-mediated tissue damage (161–163).

#### 2.2.1 Cell culture/animal model studies

PD-1 expression is closely related to inhibitory function and was considered a Treg activation marker (164, 165). In an IL-4 transgenic mouse model of AD, a gradual increase in PD-1 expression on CD4^+^ and CD8^+^ T cells was closely related to disease progression (59). Another study investigated the contribution of PD-L1 and PD-L2 in regulating the Th-type immune response using isolated APCs in three murine models with different types of inflammatory dermatitis. In the MC903-induced AD-like animal model (Th2-type model), PD-L2-deficient (PD-L2^-/-^) mice had more severe ear swelling than PD-L1-deficient (PD-L1^-/-^) mice. In that study, PD-L1 was essential for attenuating Th1- and Th17A-type immunity while PD-L2 was key in reducing Th2 immunity (60). However, another study demonstrated that PD-L2 small interfering RNA (siRNA) treatment did not inhibit the AD-like manifestations and Th2 responses in NC/Nga mice (61). These studies suggested the complex role of PD-L2 in AD-like animal models, where the reasons for the differences might be related to the use of different animal models. Therefore, the exact role of PD-L2 in AD should be explored in more studies.

#### 2.2.2 Human studies

Increased PD-1 expression on Tregs was observed in the peripheral blood of infants with moderate to severe AD (62). Several ICIs that block the PD-1–PD-L1 co-inhibitory pathway (e.g., nivolumab and pembrolizumab block PD-1 and atezolizumab blocks PD-L1) have been approved for clinical use (63). Blocking both molecules might result in irAEs, including eczema and progressive AD (166–169). In a case report, a patient with disseminated Kaposi sarcoma had a long history of AD and had never received immunotherapy. The patient’s AD improved during the pembrolizumab treatment and the overall response was good (170). The exact mechanism for the improvement remains unknown. Nevertheless, it may have been due to coincident spontaneous remission, which was unrelated to pembrolizumab treatment.

All available studies indicated that PD-1–PD-L1/PD-L2 is protective in AD, where blockage of these molecules might result in adverse effects in AD patients. However, the specific role of PD-1–PD-L1/PD-L2 in AD requires more studies for validation and to improve understanding thereof.

### 2.3 CD28 subfamily: ICOS and ICOSL

A CD28 family member, inducible T cell co-stimulator (ICOS, also known as CD278) interacts with its ligand ICOSL (CD275) and was first reported on activated human T cells (171). ICOS is expressed on activated CD4^+^ and CD8^+^ T cells and Tregs (172). The ICOS and ICOSL interactions cooperate with CD28–B7 co-stimulation to regulate immune responses and promote T cell activation and proliferation (171).

#### 2.3.1 Cell culture/animal model studies

The skin-draining lymph nodes (sdLNs) in a vitamin D3 (VitD) mouse model of AD contained increase percentages of Tregs expressing ICOS, which indicated an activated phenotype (64). However, the Treg expansion in the VitD AD-like inflammation could not counteract ongoing AD (64), which was consistent with previous findings in AD patients (173–176).

#### 2.3.2 Human studies

The peripheral blood of AD patients contained increased percentages of ICOS^+^ Tregs, which had a decreased capacity for producing IL-10 when compared to Tregs from healthy controls after restimulation, while ICOS^-^ Tregs in both groups produced very little IL-10 (65). This result indicated that despite the increased frequencies of circulating ICOS^+^ Tregs in AD, their immunosuppressive efficacy at reducing viability upon restimulation might be impaired, which in turn leads to impaired IL-10 production. In addition to the discovery of its important role in Tregs, ICOS is also key in regulating Th22 cells. The circulating Th22 subset markedly elevated ICOS expression rates in the PBMCs of a Han Chinese population with AD (66). In that study, ICOS and ICOSL expression in the lesional skin of AD patients was also significantly higher than that in the non-AD control skin. Further research revealed that ICOS^+^ Th22 cells and ICOSL^+^ B cells were closely related to disease activity and total serum IgE levels (66).

The above findings suggested that ICOS–ICOSL may be a new therapeutic target in AD and a clinical biomarker of AD disease activity. Another study of the *ICOS* gene in European children reported that none of the six evaluated *ICOS* gene variants was significantly related to the development of allergic phenotypes (67). In conclusion, the research on ICOS in AD mainly focuses on Tregs, where Treg activation might be promoted by ICOS to treat AD.

### 2.4 CD28 subfamily: BTLA and HVEM

B and T lymphocyte attenuator (BTLA), similar to CTLA-4 and PD-1, is an IgSF glycoprotein with two immunoreceptor tyrosine-based inhibitory motifs (177, 178). BTLA is induced during T cell activation and remains expressed on Th1 cells (178). The TNFR family member herpesvirus entry mediator (HVEM, or TNFRSF14) was first isolated as the receptor for herpes simplex virus 1 and is expressed on resting T cells, monocytes, and immature DCs (179). BTLA–HVEM interactions played a co-inhibitory role in T cell activation (178).

#### 2.4.1 Cell culture/animal model studies

In an *in vitro* cellular study, human CXCR5^hi^PD1^hi^ CD4 naive T (T follicular helper cells [Tfh] characteristics) were found to express elevated BTLA protein and mRNA after co-culture with thymic stromal lymphopoietin (TSLP)-activated DCs (68). The key role of TSLP in the pathogenesis of AD is well established (180). The study found a higher percentage of Tfh in AD donors compared to healthy donors (68).

There are no relevant direct reports of BTLA studies on AD so far. More studies are needed to determine the specific involvement of BTLA–HVEM signaling in AD.

### 2.5 CD2/SLAM subfamily: CD2, CD58, 2B4, CD48, and SLAM

To date, the CD2 Ig-SF comprises CD2, lymphocyte function-associated antigen 3 (LFA-3, CD58), signaling lymphocytic activation molecule 1 (SLAM, SLAMF1, CD150), SLAMF2 (CD48), SLAMF3 (Ly9, CD229), SLAMF4 (CD244, 2B4), SLAMF5 (CD84), SLAMF6 (NTBA, CD352), SLAMF7 (CRACC, CD319), SLAMF8 (BLAME), and SLAMF9 (SF2001, CD84H) (181, 182). Several receptor–ligand pairs have been reported in this family, some of which have been studied in AD. In this review, we focus on the research progress on the CD2–CD58, 2B4–CD48, and SLAM–SLAM pairs in AD. The three pairs are involved in lymphocyte activation where they induced T cell and natural killer (NK) cell proliferation, adhesion, cytokine secretion, and cytotoxicity (183–186).

#### 2.5.1 Cell culture/animal model studies

*Staphylococcus aureus* (SA) exotoxins enhanced CD48 expression in Eos when bound to CD48, causing Eos activation and signal transduction (72). The same study reported that a CD48 antagonist (neutralizing monoclonal antibody [mAb]) induced a significant reduction in SA adherence and its intracellular localization in CD48-deficient (CD48^-/-^) mice, which could be used to treat allergy (72). 2B4 has a complex role in AD, where it promoted Eos trafficking in 2B4^-/-^ mice with mild AD (induced with 2-week OVA/SEB [ovalbumin/staphylococcal enterotoxin B]). In chronic AD models (induced with 3-week OVA/SEB), 2B4^-/-^ mice had hyperdegranulated MCs, which confirmed the inhibitory 2B4 effect on MC activation (73). Further studies must clarify the complex 2B4 functions in AD. The existence of the 2B4–CD48 interaction involving both co-stimulatory and co-inhibitory signals in MCs or Eos greatly complicates the delineation of the role of 2B4–CD48 in AD (74).

#### 2.5.2 Human studies

A study of the peripheral blood of children with AD confirmed that atopy was associated with a reduced proportion of CD2^+^ cells and that the association was common in such children (69). CD2 enhances MHC–TCR interaction by binding to LFA-3 on the APC and Th1 cells express higher *CD2* mRNA levels than Th2 cells (187), thereby promoting Th1-like immunity and low CD2 expression that leads to failure to downregulate Th2 responses. The IFN-γ, granulocyte–macrophage colony-stimulating factor (GM-CSF), and IL-5 levels in the PBMCs of AD patients were reduced in the presence of anti-CD2-blocking mAb (70). That study also reported that CD28 co-stimulation restored the release cytokines in culture medium containing anti-CD2 mAbs, which suggested that CD2 and CD28 have redundant functions in T cell activation and subsequent cytokine production. Furthermore, the signaling pathway initiated by the TCR complex leading to increased IL-13 production in AD patients was largely independent of CD2 co-stimulatory signals (70). These preclinical studies demonstrated that blocking CD2 co-stimulation may exert beneficial effects on AD.

In comparison to normal non-atopic individuals, the infiltrated Eos in the skin of AD patients featured striking CD48 upregulation as opposed to its downregulation in peripheral blood leucocytes (72). The peripheral T cells of AD patients had higher *2B4* gene expression than that of non-AD participants (75).

Engagement of SLAM by an agonist mAb during the allergen-specific expansion of Th2 cell populations derived from the skin biopsies of patients with AD resulted in the generation of stable populations of IFN-γ-producing cells. SLAM-mediated reversal of the Th cell phenotype plays an important biologic role, where a new mechanism to promote Th cell differentiation was defined and a potential role for anti-SLAM mAbs for treating Th2-mediated AD was indicated (76).

The fusion protein alefacept comprises the first extracellular domain of LFA-3 (CD58) (188), where binding of the LFA-3 fragment to CD2 blocked co-stimulation and the subsequent T cell activation (189). Moreover, alefacept mediated cognate interaction between T cells and NK cells after binding to CD2 and Fcγ receptor III, which resulted in T cell apoptosis (190). In an investigator-initiated open-label pilot study, 10 patients with moderate to severe AD were treated with 12 weekly intramuscular injections of 15 mg alefacept. The treatment resulted in good therapeutic effects, which mainly included symptom improvement, reduced skin T cells, and decreased expression of inflammatory cytokines (IL-5, IL-10, IL-13, IFN-γ) (71). In another open-label study, nine patients with moderate to severe AD received 30 mg alefacept intramuscularly, where only two patients demonstrated a significant clinical response (191).

All of the above studies, where CD2, CD48, and SLAM were blocked, described favorable effects on AD. Therefore, the CD2 family might have more co-stimulatory than co-inhibitory functions in AD. However, as the functions of the CD2 family are not entirely clear, further investigations are required to identify the best possible means of exploiting them as an AD treatment. More evidence for CD2 family functions should be obtained before it can be concluded whether these molecules can be a potential target for treating AD.

### 2.6 CD226 subfamily: TIGIT and CD155

T cell immunoglobulin and ITIM domain (TIGIT, also termed WUCAM, Vstm3, or VSIG9) is a poliovirus receptor-like (PVRL) protein that belongs to the CD226 subfamily (31). TIGIT is a newly identified co-inhibitory receptor (192) expressed on NK cells, CD4^+^ T cells, CD8^+^ T cells, and Tregs in both mice and humans (192, 193). The main TIGIT ligand is CD155 (NECL-5), which belongs to the family of nectin-like (NECL) proteins (193). TIGIT and CD155 interactions not only inhibited cell proliferation but also the expression of the transcription factors T-bet, GATA3, and RORc, which specifically regulate Th1, Th2, and Th17, respectively (194).

#### 2.6.1 Human studies

A clinical analysis of 17 AD patients and 14 healthy people indicated that CD4^+^ T cells, specifically effector memory T cells and Tregs, demonstrated enhanced TIGIT expression on the patients’ PBMCs and that the proportion of TIGIT^+^ cells was correlated with AD severity (77). Further studies determined that the frequency of TIGIT^+^ cells in CD4^+^ T cells negatively correlated with the patients’ serum thymus and activation-regulated chemokine levels and IgE levels (77). In that study, TIGIT expression was increased on the AD patients’ CD4^+^ T cells to impede chronic skin inflammation, and TIGIT expression may be impaired in some AD patients, leading to the deterioration of skin inflammation (77, 195). The precise mechanism by which TIGIT expression acts in AD has not been elucidated. Blocking TIGIT shows promise for cancer therapy, where TIGIT and PD-L1 co-blockade combined with radiotherapy led to 90% cure rates in mice bearing CT26 subcutaneous tumors (196). TIGIT is an emerging immune checkpoint that inhibits immune cell responses at multiple steps of the cancer immunity cycle and constitutes a major target in cancer immunotherapy (194).

More evidence on TIGIT functions should be obtained before concluding whether it can be a potential clinical target for treating AD.

### 2.7 TIM subfamily: TIM1 and TIM4

The T cell immunoglobulin mucin (TIM) family comprises eight genes in mice, whereas only three TIM genes have been identified in humans (*TIM1*, *TIM3*, *TIM4*). In this review, we focus on TIM1, TIM3, and TIM4. TIM1 is typically expressed on Th2 cells, MCs, B cells, and NK cells while TIM4 is expressed on DCs, macrophages, and B cells (197). TIM1 is associated with the development of Th2-biased immune responses and can be selectively expressed on Th2 cells (198). The TIM family is associated with both co-stimulatory and co-inhibitory functions (199). The *TIM* gene family is involved in T cell proliferation and differentiation, which have been implicated in allergic disease (82). Only a few preclinical studies have described the role of the TIM family in AD.

#### 2.7.1 Cell culture/animal model studies

In both an OVA-sensitized AD-like mouse model and the AD-like skin lesions of NC/Nga mice, several studies reported that TIM4 expression on Langerhans-like DCs inhibited Th2 cell development and was beneficial for controlling AD (78–80).

#### 2.7.2 Human studies

Genome analysis revealed that *TIM1* exon 4 variations are associated with AD (81). A study of Australian Caucasian families and Asian families identified a novel association between AD and the major haplotype of TIM4, while there was no evidence for an association between AD and TIM3 (82). That study also suggested that genetic variants in the TIM1 and TIM4 ligand contributed to AD presentation (82).

Overall, the current studies have not determined the exact role of TIM3 in AD. TIM4 and its ligand TIM1 mainly have an inhibitory role in AD. Further research is needed to determine the best means of using TIM as an AD preventive treatment.

### 2.8 Orphan subfamily: CD160 and HVEM

A glycosylphosphatidylinositol-anchored (GPI-A) member of the Ig-SF, the co-inhibitory molecule CD160 is mainly expressed on T cells, NK cells, and all intraepithelial lymphocytes (200, 201). CD160 has a lower affinity for HVEM than BTLA (200). CD160–HVEM interactions weakened TCR-mediated signal transduction and suppressed T cell activation (202, 203).

#### 2.8.1 Human studies

Immunohistochemistry, tissue mRNA extraction, and complementary DNA sequence analysis identified a CD160^+^ T cell subset that infiltrated the inflammatory skin lesions of AD. The study also demonstrated that the engagement of CD160 enhanced the CD4^+^CD160^+^ cell proliferation induced by CD3 stimulation in ex vivo cultured T lymphocytes that infiltrated AD skin lesions (83).

The above study suggested that CD160 expression on infiltrating T lymphocytes is involved in the development of AD skin inflammation, but its exact functions *in situ* have not been elucidated experimentally.

## 3 TNF and TNFR superfamilies

Based on structure-based clustering (the TNF homology domain [THD]) revealed that the TNF superfamily is composed of several subfamilies, of which the type V (the divergent ligands [V-THD]) and L (conventional ligands [L-THD]) families members are primarily associated with co-stimulatory function, while some type L family members contribute to processes other than co-signaling functions (31, 204). TNF-SF/TNFR-SF members regulate cell differentiation, survival, and programmed death and are critical for many developmental, homeostatic, and stimulus-responsive processes *in vivo (* 205). Research on the TNFSF/TNFRSF family has greatly expanded in the past 30 years, and it is necessary to summarize the research status of this family in AD as soon as possible to better understand its role in AD to design more effective anti-inflammatory therapies.

### 3.1 Type V subfamily: OX40 and OX40L

OX40 (TNFRSF4 or CD134) is a member of the type V co-stimulation family and is mainly expressed on activated CD4^+^ T cells and CD8^+^ T cells (206). The OX40 ligand OX40L (TNFSF4, CD252) is primarily expressed on professional APCs such as DCs (206). OX40 ligation onto T cells by OX40L on APCs facilitated the effector function of T cells (46). OX40 binding to OX40L increased T cell proliferation and differentiation and cytokine production (207). OX40–OX40L signaling triggered IL-4-independent Th2 polarization, promoted TNF-α production, and inhibited IL-10 production by developing Th2 cells. In the presence of IL-12, OX40–OX40L signaling promoted the development of Th1 cells that produce TNF-α but not IL-10 (208). OX40L was a critical *in vivo* mediator of TSLP-mediated Th2 responses (208, 209).

#### 3.1.1 Cell culture/animal model studies

An *in vitro* cell study suggested that OX40L is TSLP-induced molecule on DCs that triggers inflammatory Th2 differentiation in the absence of IL-12 (84).

#### 3.1.2 Human studies

The lesional skin of AD patients contained more OX40L^+^ DCs than normal skin (85, 86). Another study reported that the skin-homing T cells in the skin of AD patients contained increased OX40 expression together with OX40 and OX40L co-localization on skin MCs (87). These results emphasized that the OX40–OX40L axis may play an important role in skin cell recruitment and activation and that blocking the OX40–OX40L signaling pathway is a potential target for AD treatment. Clinical testing of antibodies against this pathway in AD treatment demonstrated good therapeutic effects.

GBR 830 is an OX40 antagonist antibody. In a phase IIa randomized, double-blind, placebo-controlled clinical study, adults with moderate to severe AD received two intravenous injections of GBR 830 on days 1 and 29 (88). GBR 830 reduced OX40 and OX40L expression, decreased Th2, Th1, and Th17/Th22 cell numbers in skin lesions, and downregulated the mRNA expression of IFN-γ, CXCL10, IL-31, CCL11, CCL17, TSLPR, IL-23p19, IL-8, and S100A. Furthermore, GBR 830 significantly reduced the clinical symptoms of AD as compared to the placebo group. GBR 830 was safe and tolerated in 46 AD patients (88).

KHK4083 is also an OX40 antagonist antibody. In a phase I trial, injections of KHK4083 on days 1, 15, and 29 in 22 AD patients ablated OX40^+^ cells and significantly reduced AD clinical symptoms (89). Continued improvements in eczema area, severity index, and global assessment scores were observed throughout the study, with efficacy lasting until day 155 (89). A subsequent phase II clinical study enrolled 274 patients with moderate to severe AD. While the final results have not been published (49), the available results demonstrated that AD symptoms gradually improved after continuous administration of KHK4083 (>16 weeks), and long-term lasting therapeutic effects may be obtained after treatment completion.

KY1005 (SAR445229, amlitelimab) is an anti‐OX40L mAb that prevents persistent inflammation (90). In a clinical trial (NCT03161288), KY1005 demonstrated an acceptable safety and tolerability profile and novel pharmacological treatment potential in immune-mediated disorders (90). A recent study reported that combining KY1005 and mTOR (sirolimus) blockade controlled effector T cell activation effectively, preserved Treg reconstruction, and induced immune balance after transplantation (210). A phase IIa trial of KY1005 was performed successfully in AD patients (no therapeutic effect data), and a phase IIb trial has been scheduled (90).

Phase II clinical trials targeting the OX40–OX40L signaling pathway have achieved promising efficacy and safety results. Clinical studies in the future should verify the long-term safety and durability of these drugs.

### 3.2 Type V subfamily: CD27 and CD70

Type V family co-stimulatory member CD27 and its ligand CD70 have been well-characterized in humans and mice, where CD27 is expressed on naive and mature T cells, NK cells, and activated B cells (211, 212). CD70 is expressed on activated T cells, B cells, macrophages, and DCs (134). CD27 and CD70 interactions promote the expansion of antigen-specific effector/memory CD4^+^ and CD8^+^ T cells, which results in the establishment of T cell immunity (213).

#### 3.2.1 Cell culture/animal model studies

LCs were identified as the main actor in the development of AD-like symptoms in two animal models of AD (MC903-treated mice and K14-TSLP transgenic mice), although both dermal DCs and keratinocytes (KCs) contributed to the inflammatory phenotype. In the absence of LCs, these cells were unable to provoke AD disease alone (91). That study also determined that the CD70 downregulation at the LC cell surface combined with the reduced IL-12 production by LCs may be the key factor for the polarized Th2 cytokine response in the MC903-treated mice. Inhibiting LC function with CD70 might have been be a particularly effective strategy for treating AD (91).

#### 3.2.2 Human studies

An *in vitro* T cell culture study of AD patients determined that circulating Fel d 1-specific DRB1*0101-restricted CD4^+^ T cells expressed high levels of CD27, CD28, CCR7, and CD62L and correspondingly expressed low levels of tissue-specific homing receptors and Th1/2 cytokine production. Such CD4^+^ T cells with a central memory subgroup may be closely associated with long-term antigen recognition and persistent disease (92).

Nevertheless, the CD27–CD70 signaling pathways require more studies to verify their involvement in AD.

### 3.3 Type V subfamily: CD30 and CD30L

CD30 (TNFRSF8) is expressed by activated CD4^+^/CD8^+^ T cells, Tregs, and B cells, whereas its ligand CD30L (TNFSF8 or CD153) is mainly expressed on macrophages and DCs (214). CD30–CD30L interactions promoted effector and memory T cell expansion and survival and are important in humoral immune responses (215).

#### 3.3.1 Human studies

Several studies identified significantly higher serum soluble CD30 (sCD30, released by CD30^+^ cells) levels in AD patients than in non-AD controls (93–107), and sCD30 levels were positively correlated with AD disease severity (93, 95, 98, 103, 107). After treatment, the AD patients had significantly reduced sCD30 plasma levels (94, 102, 103, 216). In addition, some studies demonstrated that sCD30 concentration correlated with the disease activity and total serum IgE (101–103, 108) while others did not (105, 106).

In conclusion, CD30 appears to be a promising therapeutic target for AD. Research on CD30–CD30L in AD lacks animal model validation and relevant research on blocking antibodies, and validation and targeting studies should be encouraged.

### 3.4 Type L subfamily: LIGHT and HVEM

LIGHT (lymphotoxin-like, exhibits inducible expression, and competes with HSV glycoprotein D for HVEM; TNFSF14) is a member of the TNF family of ligands that binds three distinct members of the TNFR family: HVEM, lymphotoxin β receptor (LTβR), and soluble decoy receptor 3 (DcR3) (217). LIGHT is induced upon the activation of CD4^+^ and CD8^+^ T cells, NK cells, and immature DCs (218–220). LIGHT and HVEM are mutually regulatory on DCs and T cells, which promotes trans-signaling and minimizes cis-interactions between the two molecules when expressed in the same cell (221, 222). In the immune response process, LIGHT and HVEM engagement provides an important co-stimulatory signal for the development and survival of effector and memory T cells (223, 224).

#### 3.4.1 Cell culture/animal model studies

HVEM and LTβR are expressed on human epidermal KCs and LIGHT directly promoted TSLP expression in these cells by binding to two ligands (109). That study revealed an unappreciated activity of LIGHT on KCs and suggested that LIGHT may be an important mediator of skin inflammation in AD. An animal study demonstrated that both LIGHT-deficient mice and K14-cre HVEM^flox/flox^ (a specific lack of HVEM expression in KCs) mice exhibited minimal clinical symptoms characteristic of AD (AD-like dermatitis sensitized with HDM [house dust mite] antigens combined with SEB) (110). *In vitro*, LIGHT promoted the proliferation of normal human epidermal KCs, which the siRNA knockdown of HVEM completely prevented (110). Additionally, mice treated with anti-HVEM antibody (which neutralized LIGHT–HVEM but not LIGHT–LTβR; the antibody was administered every other day until the end of the experiment) had a markedly abrogated SCORAD (Severity Scoring of Atopic Dermatitis) index, strongly decreased epidermal thickening, and moderate albeit significant reduction in dermal thickness (110). Importantly, periostin (a clinical marker of type 2 allergic inflammatory disease) expression in the dermis was almost absent, and lower epidermal TSLP expression was observed after HVEM activity was blocked (110).

#### 3.4.2 Human studies

The peripheral serum of AD patients contained significantly elevated soluble HVEM (sHVEM) and LIGHT levels (111). Kotani and colleagues also demonstrated that plasma LIGHT concentrations correlated with IgE levels and the SCORAD index (112). In AD patients, plasma LIGHT concentrations decreased as treatment improved the symptoms (112). These results indicated that plasma LIGHT levels may be a promising biomarker for AD treatment.

These above results suggested that reagents that target either LIGHT–HVEM interactions alone or LIGHT interactions with both of its ligands may be beneficial for therapies halting and potentially abrogating AD in humans.

### 3.5 Type L subfamily: CD40 and CD40L

CD40 (TNFRSF5) is a co-stimulatory molecule that belongs to the type L family. Many APCs, such as DCs and B cells, express CD40 constitutively (225). The CD40 ligand CD40L (TNFSF5 or CD154) is expressed on activated Th cells, macrophages, B cells, and endothelial cells (225). CD40–CD40L co-stimulation activated B cells, which resulted in an immunoglobulin class switch and induced T cell activation. CD40–CD40L interactions positively modulate CD28 signaling by augmenting B7 expression in APCs. CD40–CD40L signaling is indispensable for Th2 differentiation (226, 227).

#### 3.5.1 Human studies

CD40L was significantly increased on the T cells of AD patients (228). Oflazoglu et al. demonstrated numerous CD40^+^ cells in the skin lesions and peripheral blood of AD patients and identified such cells as a positive correlation with disease severity (113). IgE levels and CD40^+^ cell numbers in AD skin lesions were correlated (113). Moreover, an *in vitro* study revealed that PBMCs isolated from AD patients had increased CD40 expression on B cells when compared with the PBMCs from non-AD donors (114). Anti-CD40 mAb distinctly increased IgE production after being added alone to the PBMCs or B cells from AD patients (114).

These findings suggested that CD40 is upregulated on APCs and that therapeutic strategies targeting the CD40–CD40L co-stimulatory pathway may benefit AD patients.

## 4 Integrin superfamilies

Integrin superfamilies are essential for both embryonic development and immunological function by binding to a wide variety of ligands, which include extracellular matrix molecules and Ig-SF members (229).

### 4.1 Leucocyte integrin subfamily: LFA-1 and ICAM-1

Also known as CD11a, CD18, or αLβ2, LFA-1 is a key T cell integrin expressed mainly in T cells and DCs (230). The cell adhesion molecule (CAM) family is a broad family that consists of intercellular adhesion molecules (ICAM-1–5), vascular CAMs (VCAMs), mucosal adhesion CAM 1 (MAdCAM-1), and activated leukocyte CAM (ALCAM) (231). In this review, we mainly focused on LFA-1–ICAM-1. LFA-1 and ICAM-1 interactions aid immune cell development and positioning, which are vital for regulating T cell activation and migration (230, 232).

#### 4.1.1 Cell culture/animal model studies

In an *in vitro* model of AD-like symptoms (TNF-α and IFN-γ stimulated KCs), 310-nm and 340-nm ultraviolet light-emitting diodes (UV-LEDs) effectively inhibited STAT1 signaling and suppressed inflammation by downregulating ICAM-1. That study suggested that 310-nm and 340-nm UV-LED phototherapy may be a therapeutic strategy for AD (115). Moreover, in an atopic-like dermatitis model in NC/Nga mice, anti-LFA-1 mAb inhibited the development of skin lesions, IgE production, and lymphocyte cytokine production (IL-4 and IFN-γ), which most likely occurred by inhibiting antigen presentation (116).

#### 4.1.2 Human studies

Increased ICAM-1 and VCAM-1 expression was demonstrated in skin biopsies from chronic and acute AD lesions (117). ICAM-1 was strongly expressed on KCs in lesional atopic eczema but was not a suitable marker of actual disease activity (118). Furthermore, AD patients had significantly higher soluble ICAM-1 (sICAM-1) levels than people without AD (119–121). A study that used intravenous immunoglobulin (IVIG) to treat severe intractable AD in children reported that ICAM was significantly reduced after the IVIG treatment and that the determination of ICAM-1 levels may be useful for monitoring the disease activity of AD in childhood (122). Another study demonstrated that tacrolimus-treated AD patients had significantly reduced ICAM-1 expression while hydrocortisone-treated patients did not. Inhibiting ICAM-1 expression may represent another selective mechanism of topical tacrolimus in AD treatment (123).

Efalizumab is a humanized mAb specific to LFA-1 that was originally approved for treating psoriasis and has demonstrated some therapeutic value in AD (233). In one case, efalizumab successfully improved the clinical symptoms in children and adults with severe AD, with outstanding improvement in clinical symptoms after 6 months of treatment (124). Hassan et al. reported a case of severe atopic eczema treated with efalizumab monotherapy, which resulted in a notable reduction in inflammatory cell infiltration in the skin lesions (125). A concurrent report demonstrated that efalizumab therapy produced a striking clinical improvement in most participants with severe AD, such as improvement of erythema and excoriation and decreased pruritus levels. Takiguchi et al. considered efalizumab a potential alternative to the systemic immunosuppressants currently used for AD, but recommended that its safety and efficacy should be tested in randomized double-blind placebo-controlled studies before it could be recommended for routine use in this patient population (126). Nevertheless, a subsequent retrospective study of psoriasis involving 11 efalizumab-treated patients cast doubt on these results, where only a few patients with severe AD responded to efalizumab at the recommended standard dose (0.7 mg/kg initially followed by 1 mg/kg weekly) (127).

In conclusion, targeting the LFA-1–ICAM-1 signaling pathway shows promise for treating AD. Nonetheless, more clinical studies are needed for further testing.

## 5 Safety of targeting co-signaling molecules in AD therapy

irAEs are common in ICI cancer treatment. While the mechanism remains unknown, it is related to the ICI mechanism and individual differences (234). irAEs typically occur early in treatment and are dose-dependent (235–237). Melanoma treatment with higher ipilimumab doses was followed by higher irAE incidence and severity (237). AD/eczema, which accounts for approximately 17% of irAE, is the most common characteristic (155). ICI-related cutaneous adverse reactions are mainly mild to moderate and easily treated. Understanding the potential for skin toxicity and the timely identification and management of specific irAEs is essential. Early responses to manage irAEs are beneficial to cancer treatment maintenance.

The above studies demonstrated that the co-inhibitory blockers used to treat cancer can induce irAEs, which suggested that blocking the co-stimulatory signal molecules may be a promising AD treatment. Targeting the co-stimulatory molecules OX40, OX40L, CD58, and LFA-1 to treat moderate to severe AD achieved specific clinical efficacy and demonstrated acceptable safety and tolerability. The incidence of drug-associated irAEs in AD clinical studies with GBR 830 (OX40 antagonist antibody) treatment was 62.9%, where most irAEs were of mild to moderate intensity (88). Severe irAEs accounted for 3.2% of the total cases. The investigators did not determine whether the serious irAEs were related to the treatment (88). In a phase I clinical trial, targeting treatment with KHK4083, another OX40 antibody, also demonstrated mild or moderate irAEs. All irAEs did not affect the KHK4083 treatment course (89). A phase IIa clinical trial of KY1005 targeting OX40L demonstrated a good safety profile (90). Similar results were observed in clinical trials of alefacept (targeting CD58) (71, 191) and efalizumab (targeting LFA-1) (124–127).

Targeting treatment at co-signaling molecules may be a promising future approach. More large-sample, long-term clinical studies would aid understanding of their safety.

## 6 Future and clinical perspectives

Co-stimulatory and co-inhibitory receptors play key roles in T cell biology and act as secondary signals to determine the functional outcome of TCR signaling. The classical definition of T cell co-stimulation continues to evolve through the identification of novel co-stimulatory and co-inhibitory receptors, the biochemical characterization of their downstream signaling events, and the characterization of their immune function. After decades of development, Chen et al. reported that co-stimulatory and co-inhibitory receptors exhibit highly diverse and complex expression, structure, and function, and their function is largely influenced by the environment (31). The current research status suggests that the co-signaling pathways in AD are clearly a huge family. Various co-stimulatory and co-inhibitory pathways affect and shape the complex immune inflammation in AD. Many signaling pathways play a role in both co-stimulation and co-inhibition due to the different cell types in which they are expressed. For example, interaction between HVEM expressed on T cells with LIGHT on APCs exerted a stimulatory effect while the interaction between HVEM expressed on APCs and CD160/BTLA expressed on T cells exerted a suppressive effect. In addition, LIGHT has other receptors and HVEM has more than just two ligands. This is only one example of co-signaling molecules, and more complex roles and more receptors exist (31).

Several co-stimulatory signaling targeting drugs mentioned in this review are in the clinical research stage. The drugs (GBR 830, KHK4083, KY1005) targeting the OX40–OX40L co-stimulatory pathways are clinically used in AD treatment (88–90). Phase I clinical trials of these drugs demonstrated good therapeutic effects with only some minor adverse effects. Phase II clinical trials of KHK4083 and KY1005 have also demonstrated great promise. Although the phase I clinical trial of efalizumab targeting the LFA-1–ICAM-1 co-stimulatory pathway demonstrated good therapeutic effects, its adverse events cannot be ignored. It is worth noting that efalizumab was withdrawn from the US psoriasis treatment market in 2009 due to the risk of progressive multifocal leukoencephalopathy (238). Accordingly, there is a need to balance the advantages and disadvantages of this pathway for future use in AD treatment. Targeting antibodies against the CD2–CD58 signaling pathway also demonstrated good therapeutic effects, but the small number of clinical samples and lack of long-term treatment results mean that conducting more clinical trials in the future would be worthwhile (191).

Other targeting proteins and antibodies have demonstrated certain therapeutic effects in mouse animal experiments or *in vitro* cell experiments. Among them, targeting the CD40–CD40L signaling pathway is noteworthy. Conventionally, it is believed that blocking the co-stimulatory signaling pathway weakens the co-stimulatory effect and reduces the inflammatory indicators. However, strong lymphocyte proliferation and lgE expression expansion were observed *in vitro* cell experiments (114). It has been speculated that differences in CD40 expression cause the reverse effect of anti-CD40 mAb in AD. These findings echo the expansiveness and complexity of the aforementioned co-signaling pathways, which can contribute unequally to the effects of stimulation or inhibition due to differences in the expression of the same ligand on different cells and can be affected by the model system used. Therefore, the resulting immune response appears to be the dominant side effect of the interaction of each signaling pathway. Researching and exploring co-stimulatory signaling pathways is challenging. The PD-1–PD-L1/PD-L2 signaling pathway is a notable co-inhibitory signaling pathway. PD-1–PD-L1/PD-L2 targeting in cancer has demonstrated great scientific research progress, and related ICIs are widely used in clinical treatment and trials, which have recorded remarkable therapeutic effects (162, 239). Unfortunately, this immune checkpoint-targeted therapy causes irAEs (240). Interestingly, most skin irAEs tend to be psoriasis, acneiform rash, vitiligo-like lesions, and autoimmune skin diseases (bullous pemphigoid, dermatomyositis, alopecia areata), while AD is rare (48). Consistent with this, treating Kaposi sarcoma with a PD-1 blocking antibody (pembrolizumab) improved AD symptoms. However, siRNA treatment against PD-L2 was ineffective in NC/Nga mouse experiments. These results suggested that more research and validation of this co-inhibitory pathway are needed in the future.

Based on the above summary, many problems and limitations persist in current research on the co-signaling pathways in AD. The roles of certain co-stimulatory and co-inhibitory signaling pathways in AD obviously deviate from the expected results, and research is superficial, where it involves (only changes in clinical symptoms and downstream cytokines). An in-depth study of the specific mechanism is crucial for developing and improving AD targeted therapy strategies in the future. Moreover, whether there are more co-stimulatory and co-inhibitory receptors or new receptors or ligands of known molecules to be discovered cannot be ruled out, and there are more aspects worth exploring.

Efalizumab was discontinued in psoriasis treatment due to adverse effects. Therefore, how can the adverse effects caused by targeting signaling pathways be minimized or even eliminated? While therapeutics using targeting co-signaling molecules offer desirable outcomes, they also present unique challenges. Can more specific targeted therapies that only act on the skin be developed? Fortunately, specific antibodies can be targeted to specific tissues and organs through the targeted delivery of engineered viral vectors or nanoparticles (241, 242). It is possible that co-signal targeting antibodies against AD may be further optimized in the abovementioned direction in the future.

In this article, we repeatedly mentioned combined treatment methods, such as anti-OX40L and anti-IL-4 combined, which achieved good results. Moreover, the synergistic inhibition of anti-PD-1 and anti-TIGIT yielded the best results for inhibiting T cell activity (243). However, combination therapy with anti-CTLA-4 and anti-PD-1 drugs was associated with a higher frequency of toxicity (244). Given the unsatisfactory effects yielded by the combined application of some co-signaling molecules, more research is needed to explore mutual promotion and mutual exclusion and to obtain effective combination solutions that benefit both research and treatment. Another option is the possibility of combining other conventional drugs for AD based on the application of biological agents to obtain the best therapeutic effect.

Given that there are no guidelines currently available, perhaps the development of synergistic signaling pathway-targeted therapies in the future can enable greater selectivity in AD treatment guidelines. Furthermore, how can specific immune cell populations be targeted more precisely? As co-signaling molecules are widely expressed on various immune cell types, targeting any pathway can yield unexpected results, such as the aforementioned biased results of the anti-CD40–CD40L signaling pathway, which is both the appeal and problem for the immune system. This raises the question of whether it might be possible to develop treatments that target a specific cell type and lead to more precise treatment and resolve the recurring episodes of AD that have long troubled doctors and patients.

## 7 Conclusions

This review not only discusses the current research progress of targeted therapy of co-signaling molecules in AD but also presents unresolved issues and existing limitations. Several biologics targeting co-signaling molecules have demonstrated promising results in AD patients. Blockade of the T cell co-stimulatory pathways may be a promising therapeutic target in AD, and inhibiting the T cell co-suppressive signaling pathways may produce irAEs. Given the significant impact of the co-stimulatory and co-inhibitory pathways in AD, there are plausible reasons and a clear motivation to expand our understanding of these co-signaling molecules in AD and determine their full effect. We believe that targeting the common signaling pathways for AD shows strong promise.

## Author contributions

CZ wrote the manuscript; YS and YZ critically revised the manuscript. All authors contributed to the article and approved the submitted version.
